# Serial Shunt Evaluation Reveals Limitations of Contemporary Screening Studies for Patent Foramen Ovale

**DOI:** 10.1016/j.jcin.2020.01.210

**Published:** 2020-05-25

**Authors:** Pok-Tin Tang, Thomas Cahill, Peter M. Rothwell, Oliver J. Ormerod, Matthew J. Daniels

Percutaneous closure of a patent foramen ovale (PFO) reduces stroke in appropriately selected patients (1). PFO screening typically employs agitated saline injection into the brachiocephalic vein, which drains via the superior vena cava (SVC) to the right atrium. Bubbles bypassing the lungs are detected by echocardiography or transcranial Doppler (2). However, venous thrombus preferentially forms in the lower limbs, entering the heart via the inferior vena cava (IVC). Anatomic structures of the right atrium preferentially direct IVC flow to the PFO (3). Often IVC flow predominates, washing arm injection bubbles away from the PFO.

Subgroup analyses of the PFO closure (PFOc) trials by shunt magnitude do not show a clear treatment advantage for small shunts (1). However, the trials lacked power to address this. To determine whether certain subgroups can be managed with medical therapy alone, it becomes important to understand whether the historical limitations of the SVC-delivered screening bubble study (4,5) have a meaningful impact on young cryptogenic stroke cohorts referred for PFOc.

Early in our PFOc experience, we encountered catheter perforation when pushing on a closed fossa ovalis. We subsequently included an on-the-table bubble study (8-ml saline, 1-ml blood, 1-ml air, repeated up to 3 times) allowing sequential investigation at rest, with a sniff or full Valsalva from both SVC and IVC directly using a single 6-F Sones 1 catheter advanced from a right femoral venous puncture as a routine part of the PFOc process. We delay heparin administration until PFO is confirmed by a visible shunt, or subsequent mechanical probing during intracardiac echo (ICE)-guided PFOc. This invasive dataset was obtained a few months after the positive screening transthoracic arm bubble study that triggered referral for closure. A total of 288 consecutive patients treated in our center between January 4, 2011 and March 22, 2017 (average age 47.1 years, 57% male, 73% following definite stroke, 4.2% smoking history, 1.7% diabetic, 2.1% ischemic heart disease, 1% pulmonary disease) produced a complete study dataset for 244 patients. Shunts were classified by magnitude into 4 groups: none, mild (<10 bubbles), moderate (>10 bubbles), or large (clouds of uncountable bubbles), from the IVC and SVC, paralleling contemporary trial reports and bubble counting methodology (Table 1); shunts at rest were graded higher than shunts requiring provocation.

**Table 1:** Summary Data of On-Table IVC and SVC Bubble Contrast Studies in a 244 PFO Patient Cohort

		SVC Shunt Grade			
		None	Mild	Moderate	Large
IVC Shunt Grade	None	40	2	1	0
	Mild	34	27	4	2
	Moderate	20	21	30	3
	Large	13	18	7	22

Values are n.

Shunts were studied from the SVC and IVC before closure. They were graded as absent (none), mild, moderate, or large. Shunts where IVC flow predominates are colored in shades of green, balanced shunts in shades of orange, and SVC flow dominant in red. Although SVC screening studies are preferred in contemporary practice, SVC dominant flow is the exception (4.9%, 12 of 244) rather than the rule.

IVC = inferior vena cava; PFO = patent foramen ovale; SVC = superior vena cava.

IVC and SVC shunt magnitude is matched in 48.8% of patients (119 of 244). However, 16.4% of patients (40 of 244) with positive screening arm studies have no demonstrable on-table shunt from either SVC or IVC injection. Of this group, 16 patients lacked a PFO when the fossa ovalis was probed. There is a disparity (chi square = 39.8, df = 1; p < 0.0001) in negative bubble studies from the SVC, 43.9% of patients (107 of 244), compared with the IVC (43 of 244, 17.6%). Importantly 29.5% of patients (72 of 244) with absent or mild SVC shunts have moderate/severe IVC shunts. The IVC shunt exceeded the SVC shunt in 46.3% of patients (113 of 244). Patients with large SVC shunts (11.0%, 27 of 244) almost invariably had matched large or moderate IVC shunts (92.6%, 25 of 27). Sensitivity and specificity for SVC injections was 60.0% and 100% compared with 88.2% and 100% for IVC injections.

We believe this is the first study reporting serial physiological testing of PFO shunting in patients being evaluated for closure. Although prior work demonstrates increased sensitivity of repeated contrast injections (4) (the diagnostic yield plateaus after 10 injections) or IVC injections (5), different protocols were followed in the randomized PFOc trials (typically 3 to 5 injections via the arm). Contemporary practice may not match trial standards.

We highlight 3 points; first, our results suggest that almost one-half the patient population (here, 43.9%) will not have a demonstrable SVC shunt on serial evaluation on the basis of 3 injections including provocation. Thus, isolated screening assessments following suspected paradoxical embolism may be inadequate. Second, although a large SVC shunt predicts a significant IVC shunt, the converse is not true; most patients with large IVC shunts do not have large SVC shunts. Third, the IVC shunt grade is often (here, 46.3%) underestimated by the SVC shunt. It may, therefore, be unwise to restrict PFOc to certain patient groups on the basis of SVC studies with limited reproducibility and accuracy for the IVC flow, which preferentially carries the embolic material causing cryptogenic stroke. Indeed, we now undertake IVC screening for patients with compelling stroke histories (e.g., Valsalva, immobilization, etc.) or repeated presentations, before concluding noninvasively that a PFO is absent.

There are limitations to this retrospective single-center study. Right-sided filling pressures may be reduced by the expected pre-procedure fast and sedation; on-table studies are supine rather than screening, which is performed at 30°. These factors may increase the likelihood of a negative second bubble study. Equally, the image quality of ICE and immediate large-volume contrast delivery to the IVC or SVC may bias results in favor of shunt detection.
